# Book Notices

**Published:** 1894-04

**Authors:** 


### Book Notices.

---

REACTIONS. A Selection of Organic Chemical Preparations Important to Pharmacy in Regard to their Behavior to Commonly used Reagents. By F. A. Flückiger, Ph.D., M.D. Authorized English Edition. Translated, Revised and Enlarged by J. B. Nagelvoort, Analytical Chemist to the Pharm. Chem. Laboratory of Parke, Davis & Co. (With Portrait and Autograph of the Author.) Bound in cloth, large octavo; price, $2. Geo. S. Davis, Medical Publisher, Detroit, Mich.

This is a carefully prepared book of 154 large octavo pages, printed on extra heavy paper, and very stylish in appearance. A list of the principal reagents is given, followed by a concise description of the experimental tests made by the author and others, and the reactions resulting from the applications of these tests. The tests and the method of applying them to the various chemicals under consideration, are purposely of the most simple, and without going into theoretical explanation, which would unnecessarily enlarge the scope of the volume; the author aims at practical information only. Some of the reactions are here described for the first time, and the author frankly acknowledges that they are not yet fully understood.

In translating the volume from the German the translator has not attempted a verbatim translation, but rather a revised and enlarged edition of it. He has further added to the value of the original work by giving the result of his own experiments where the statements of the author were not concordant with those of other leading investigators.

The book is an excellent one and will rank high in pharmaceutical literature. H.

A Clinical Text-Book of Medical Diagnosis for Physicians and Students Based on the Most Recent Methods of Examination. By Oswald Vierordt, M. D., Professor of Medicine at the University of Heidelberg, formerly Privat-Docent at the University of Leipzig; later, Professor of Medicine and Director of the Medical Polyclinic at the University of Jena. Authorized translation, with additions, by Francis H. Stuart, A.M., M.D., member of the Medical Society of the county of Kings, New York; Fellow of the New York Academy of Medicine; Member of the British Medical Association, etc. Third revised edition. 700 pages, with one hundred and seventy-eight illustrations, many of which are in colors. Price, cloth, $4; sheep, $5; half Russia, $5.50. W. B. Saunders, Publisher, 925 Walnut St., Philadelphia.

The first edition of this splendid work was issued in 1888, and in the short period of six years the third edition has become necessary. It has been translated into the English, Russian, Italian and Spanish languages. This fact attests the high esteem in which the first and second editions of the book have been held, and now that we have a third edition, with corrections of the small errors found in the previous editions, and containing the latest information bearing on the diagnosis of diseases, thus bringing the work fully up to date, there is no reason why its popularity will not be vastly increased.

Prof. Vierordt, having been a teacher of diagnosis in some of the leading schools of Europe for the past ten years, is eminently qualified to prepare a book on this subject, of the highest order of merit, and we doubt if this one has a superior anywhere.

The book is divided into eight chapters, with the following general heads:

Chapter I, Introduction; Chapter II, Examination of patients; Chapter III, General examination; Chapter IV, Examination of the respiratory apparatus; Chapter V, Examination of the circulatory apparatus; Chapter VI, Examination of the digestive apparatus; Chapter VII, Examination of the urinary apparatus; Chapter VIII, Examination of the nervous system. It also has an appendix treating of "Lanyngoscopic Examinations of the Larynx," "Paralysis of the Muscles of the Larynx," "Examinations with the Ophthalmoscope," and "Bacteria which come under Consideration in the Diagnosis of Internal Diseases."

The index covers ninety-two pages, and is the most complete that we have ever seen in any book.

The book is well arranged, and splendidly written, and the translator has performed his labors very satisfactorily. It will be a source of gratification to many of the profession of Texas to know that the translator acknowledges the valuable services of Prof. George Dock, formerly of Galveston, in "the correction of many slight errors and suggestions of improvement." Prof. Dock was formerly a student of Prof. Vierordt, and uses this work as a text-book in his classes. H.

---

HERNIA: ITS PALLIATIVE AND RADICAL TREATMENT IN ADULTS, CHILDREN AND INFANTS. By Thomas H. Manley, A. M., M. D., Visiting Physician to Harlem Hospital, Consulting Surgeon to Fordham Hospital, Member of New York Academy of Medicine, American Medical Association, New York State and County Medical Associations, etc., etc. In one volume of 230 pages, profusely illustrated. Bound in cloth. Published by the Medical Press Co., Limited, 1725 Arch St., Philadelphia, Pa.

In view of the many changes that have been made in the treatment of hernia during the past twenty years, and the diversity of opinions held by leading men in the profession at this time, a book, such as this, written by one holding conservative views on the subject, is almost a necessity, and is a valuable addition to our list of medical works. Dr. Manley advocates a middle ground, opposed on the one hand to those who object to operative interference, except in cases of strangulated hernia, and to those on the other extreme who advise a surgical operation for the radical cure of all cases of hernia. He points out the serious injuries that may be done by trusses and other surgical appliances for the purpose of pressure, especially in young children. The book is well written throughout, and will meet with the approval of the majority of conservative surgeons.

H.

---

TRANSACTIONS OF THE SOUTHERN SURGICAL AND GYNECOLOGICAL ASSOCIATION. Volume V, the Session held at Louisville, Kentucky, November 16, 17 and 18, 1892. Published by the Association: 1893.

This volume is a credit to any medical organization, and the publishing committee, consisting of Dr. W. E. B. Davis, of Birmingham, Alabama; Dr. W. B. Rogers, of Memphis, Tennessee, and Dr. V. O. Hardon, of Atlanta, Georgia, deserves much credit for the excellent style in which the transactions are gotten out. The volume of 434 pages, including the index, contains many valuable papers on surgical and gynecological subjects, contributed by men of intelligence and experience. The paper on "Combined Surgical and Electrical Treatment of the Brain in Epilepsy," by our Dr. B. E. Hadra, of Galveston, is in this volume of the Transactions. This is a valuable contribution to medical literature, and we think adds much to the worth of this excellent volume of Transactions. H.

A PRACTICAL TREATISE ON MATERIA MEDICA AND THERAPEUTICS. By Roberts Bartholow, M. A., M. D., LL. D. Eighth edition, revised and enlarged. New York: D. Appleton & Co. 1893.

The eighth edition of this book has come to our exchange, and by oversight has not been noticed earlier. We can but refer to it in the very highest terms. It is a work that has been in the hands of students and physicians for the past seventeen years, and during that time eight editions have been issued, which bespeaks its patronage and the efforts of the author to keep it well up to all the recent advances in the medical world.

We must especially refer to the lengthy physiological action given to each drug, which is the true basis of a proper understanding of their action. This coupled with the author's many years experience as a professor of the practice of medicine and materia medica and therapeutics respectively, and his large private practice, makes this book one of the most complete, thoroughly practical and reliable works of the kind of equal size now extant. All changes deemed advisable have been added since the revision of the pharmacopœia. M. M. S.

---

A SYLLABUS OF LECTURES ON THE PRACTICE OF SURGERY. Arranged in conformity with the American Text-Book of Surgery. By N. Senn, M. D., Ph. D., LL. D., Chicago, Professor of the Practice of Surgery and Clinical Surgery in Rush Medical College; Professor of Surgery in the Chicago Polyclinic; Attending Surgeon to Presbyterian Hospital; Surgeon-in-Chief St. Joseph's Hospital; President Association of Military Surgeons of the United States; Ex-President American Surgical Association, etc., etc. W. B. Saunders, Publisher, 925 Walnut Street, Philadelphia.

This book is prepared for the especial use of teachers of surgery, in order that they may have a guide in presenting the various subjects to their classes in a systematic and practical manner, and for students of surgery that they may be able to memorize the essentials, as here presented, in the most concise form possible. The arrangement of this syllabus is excellent, and it goes without saying that the book is first class in every respect, as Dr. Senn does nothing by halves. It will prove valuable to every surgeon, or student of surgery. H.

---

A CHAPTER ON CHOLERA FOR LAY READERS. History, Symptoms, Prevention and Treatment of the Disease. By Walter Vought, Ph.B., M.D., Medical Director and Physician-in-Charge of the Fire Island Quarantine Station, Port of New York; Fellow of the New York Academy of Medicine, etc. Illustrated with Colored Plates and Wood Engravings. In one small 12mo volume, 110 pages. Price, 75 cents net. Philadelphia: The F. A. Davis Co., Publishers, 1914 and 1916 Cherry Street.

This little book, as its name indicates, is prepared for the especial use of the laity, but there are many points in it, especially in the chapter on "Prevention," that might be read with profit by a large number of the medical profession. The book will answer the purpose for which it was written, splendidly, and we do not hesitate to commend it. If a copy of this book could be placed in the hands of every family, when an epidemic of cholera is threatening, it would prove of much assistance to sanitary officers and physicians, and might be the means of saving many lives. H.

---

THE SURGICAL ANATOMY AND SURGERY OF THE EAR, by Albert H. Tuttle, M. D., S. B. (Harv.) of Cambridge, Mass. Member of the Massachusetts Medical Society; Member of the American Medical Association, etc., etc., etc. With twenty-eight original illustrations, reproduced from the writer's drawings from nature. Price, paper, 25 cents; cloth, 50 cents. Geo. S. Davis, publisher, Detroit, Mich.

This is an excellent monograph on the surgical anatomy of the ear, together with many very valuable new illustration, and instructions for the surgical treatment of ear affection. The author does not claim that this little book includes all the important questions on the subject of aural surgery and anatomy, but he has tried to select those which offer the greatest from a surgical standpoint.

---

A SYSTEM OF LEGAL MEDICINE. A Complete Work of Reference for Medical and Legal Practitioners, by Allan McLane Hamilton, M. D., of New York, and Lawrence Godkin, esq., of the New York Bar, assisted by thirty Collaborators of recognized ability. In two royal octavo volumes of about 700 pages each. Fully illustrated. E. B. TREAT, 5 Cooper Union, New York.

The great need of a standard American work on medical jurisprudence has long been felt; and this work gives abundant promise of being just what the medical and legal profession have so long wanted. Every department will be thoroughly and reliably treated.

---

EDWARD BOK'S successful article in the January "*Cosmopolitan*" on "The Young Man in Business" has been reprinted in a tasteful and handy booklet form at 10 cents, by The Curtis Publishing Company, of Philadelphia. To this reprint Mr. Bok has added some 14 pages of editorial matter answering "Three Uncertain Young Men."

